# Hematological complications in solid organ transplant recipients with telomere biology disorders: a narrative review

**DOI:** 10.3389/fimmu.2025.1718107

**Published:** 2026-01-15

**Authors:** François M. Carlier, Thomas Planté-Bordeneuve, Antoine Froidure, Carlos Graux, Marie-Astrid van Dievoet, Coline H. M. van Moorsel, Thijs W. Hoffman

**Affiliations:** 1 Pole of Lung, Nose and Skin (LUNS), Institut de Recherche Expérimentale et Clinique (IREC), Université Catholique de Louvain, Brussels, Belgium; 2 Department of Pneumology, CHU Mont-Godinne UCL Namur, Yvoir, Belgium; 3 Lung Transplant Centre, CHU Mont-Godinne UCL Namur, Yvoir, Belgium; 4 Department of Pneumology, Cliniques Universitaires Saint-Luc, Brussels, Belgium; 5 Department of Hematology, CHU Mont-Godinne UCL Namur, Yvoir, Belgium; 6 Laboratory Department, Cliniques Universitaires Saint-Luc, Brussels, Belgium; 7 ILD Center of Excellence, Sint Antonius Hospital, Nieuwegein, Netherlands

**Keywords:** hematology, lung transplantation, review, short telomeres, solid organ transplantation, TBD, telomere biology disorders, telomeres

## Abstract

Telomeres are repetitive nucleotide sequences at the ends of chromosomes that preserve genomic integrity. Defects in telomere maintenance mechanisms lead to premature telomere shortening, resulting in cellular senescence, apoptosis, and organ dysfunction, collectively termed telomere biology disorders (TBDs). Short telomere length is associated with an increased risk of end-stage fibrotic disease of the lung and/or liver, which may necessitate lung or liver transplantation. Beyond pulmonary and hepatic involvement, TBDs can also affect cardiac and renal function. Importantly, the bone marrow function is often also compromised, which can significantly influence transplant outcomes. Although evidence remains scarce, particularly in non-lung solid organ transplant recipients, post-transplant immunosuppressive therapy, typically including corticosteroids, calcineurin inhibitors, and cell cycle inhibitors, may exacerbate the underlying hematopoietic fragility in TBD patients. Hematological complications may result from both the intrinsic TBD and the additive myelotoxic effects of immunosuppressive agents (e.g., azathioprine, mycophenolate mofetil) or anti-infectious prophylaxis (e.g., trimethoprim-sulfamethoxazole, valganciclovir). Early recognition of TBDs prior to transplantation is essential. Assessment of telomere length and genetic testing should be considered in at-risk candidates, particularly those with early-onset pulmonary fibrosis, unexplained cytopenia, cryptogenic liver disease, or a family history suggestive of TBD. A multidisciplinary approach involving pulmonology, hepatology, hematology, and transplant specialists is crucial to optimize patient selection, perioperative management, and post-transplant care. This review summarizes current knowledge on hematological complications following solid organ transplantation in TBD patients and describes expert-opinion strategies for the pre-transplant evaluation and post-transplant management of these high-risk individuals.

## 1 Introduction

Telomeres are non-coding DNA fragments at the ends of chromosomes that protect the genome from degradation and act as a natural “biological clock”, as they shorten after each cell division, ultimately leading to cell senescence. Natural telomere shortening is partially compensated by the enzymatic telomerase complex, although the expression of telomerase-related genes is highly variable in different organs and cell types (1). Telomere biology disorders (TBDs) are mono- or multi-organ diseases characterized by defects in telomere maintenance, often associated with premature telomere shortening and cellular senescence. Telomerase dysfunction and telomere attrition affect the ability of progenitor cells to self-renew (2), with varying consequences across different organ systems: bone marrow failure (aplastic anemia), cutaneous manifestations such as early hair greying, liver cirrhosis, or pulmonary fibrosis are the most typical presentations of TBD (3).

Most TBD cases involve germline mutations in telomere-related genes (TRG), such as *TERT*, *TERC*, *RTEL1* and *PARN*, associated with telomerase complex dysfunction. However, in a large proportion of patients the underlying genetic background remains unknown, as no variant is identified in some patients with a typical clinical phenotype and a substantial part of identified variants is of (yet) unknown significance. In idiopathic pulmonary fibrosis, the most frequent TBD, up to half of individuals display short telomeres (4, 5), while a pathogenic mutation in TRG is only found in 10%-15%.

TBDs are increasingly recognized as an important risk factor for hematologic and immunologic dysfunction: having short telomeres is associated with poorer outcomes in septic shock (6), COVID-19 infection (7) and increased risk of hematological complications related to immunosuppressive drugs (8, 9). This latter point is crucial in the setting of solid organ transplant recipients as it could impact post-transplant survival. While the impact of TBDs has been studied in some aspects of lung transplant recipients, especially those with interstitial lung disease (10), their impact on post-transplant outcomes across other organ systems remains poorly understood.

In this review, we will synthesize current knowledge on the hematological complications in solid organ transplantation recipients with TBDs and highlight the underlying mechanisms, both from a genetic and biological perspective. Finally, we will discuss the clinical implications for transplant eligibility, management, and long-term outcomes.

## 2 From genetic mechanisms to phenotype

### 2.1 Genetic basis of TBDs

Known telomere maintenance proteins include components of the telomerase complex, shelterin, the Ctc1-Stn1-Ten1 (CST) complex, DNA polymerases and factors required for hTR maturation (11–13). Mutations in 19 genes encoding telomere maintenance proteins have been found to underlie different types of TBDs, and telomere gene mutations can lead to disease manifestations in different organ systems. **Table 1** summarizes the currently recognized genes implicated in telomere maintenance, their molecular functions, associated TBD entities, and typical inheritance patterns.

**Table 1:** Telomere maintenance genes with mutations described in TBD patients with their reported disease manifestations and affected alleles.

Gene	Function	TBD entity	Alleles affected	References
*TERC*	Part of telomerase complex	DKC, Hoyeraal-Hreidarsson syndrome, hematological disease, pulmonary fibrosis, liver disease	Mono-allelic	(3, 14–32)
*DKC1*	Part of telomerase complex, stabilization of hTR	DKC, Hoyeraal-Hreidarsson syndrome, hematological disease, pulmonary fibrosis, liver disease	X-linked	(20, 33–72)
*NOP10*	Part of telomerase complex, stabilization of hTR	DKC	Bi-allelic	(73)
		Pulmonary fibrosis, hematological disease	Mono-allelic	(74, 75)
*NHP2*	Part of telomerase complex, stabilization of hTR	DKC, Hoyeraal-Hreidarsson syndrome	Bi-allelic	(76–79)
		Pulmonary fibrosis, liver disease	Mono-allelic	(75, 77, 80)
*TERT*	Part of telomerase complex	DKC, Hoyeraal-Hreidarsson syndrome	Bi-allelic	(53, 81–86)
		DKC, hematological disease, pulmonary fibrosis, liver disease	Mono-allelic	(3, 14, 21, 22, 24–28, 52, 87–104)
*NAF1*	Part of telomerase complex	DKC, hematological disease, pulmonary fibrosis, emphysema, liver disease	Mono-allelic	(105)
*TINF2*	Part of shelterin	DKC, Hoyeraal-Hreidarsson syndrome, Revesz syndrome, hematological disease, pulmonary fibrosis, liver disease	Mono-allelic	(80, 106–125)
*POT1*	Part of shelterin	Coats-plus syndrome	Bi-allelic	(126)
		Pulmonary fibrosis, liver disease	Mono-allelic	(127)
*ACD*	Part of shelterin	DKC, Hoyeraal-Hreidarsson syndrome	Bi-allelic	(128–130)
		Pulmonary fibrosis, hematological disease, liver disease	Mono-allelic	(130–132),
*STN1*	Part of CST-complex	Coats-plus syndrome	Bi-allelic	(133–136)
*CTC1*	Part of CST-complex	DKC, Coats-plus syndrome	Bi-allelic	(136–146)
		Hematological disease, pulmonary fibrosis	Mono-allelic	(147, 148)
*PARN*	Exoribonuclease involved in hTR maturation	DKC, Hoyeraal-Hreidarsson syndrome, Pulmonary fibrosis	Bi-allelic	(149–156)
		Pulmonary fibrosis	Mono-allelic	(27, 75, 125, 155, 157–160)
*ZCCHC8*	Involved in hTR maturation	Pulmonary fibrosis, hematological disease	Mono-allelic	(32, 161, 162)
*RTEL1*	DNA-helicase that facilitates telomerase function	DKC, Hoyeraal-Hreidarsson syndrome, hematological disease	Bi-allelic	(163, 164)
		DKC, pulmonary fibrosis, hematological disease, liver disease	Mono-allelic	(75, 125, 165)
*RPA1*	Facilitates telomere replication	DKC, hematological disease, pulmonary fibrosis	Mono-allelic	(166)
*DCLRE1B*	Required for telomere stability	DKC, Hoyeraal-Hreidarsson syndrome	Bi-allelic	(167)
*POLA1*	DNA-polymerase required for telomere replication	DKC, hematological disease, pulmonary fibrosis	X-linked	(168)
*POLA2*	DNA-polymerase required for telomere replication	Coats-plus syndrome	Bi-allelic	(169)
*WRAP53*	Facilitates telomerase localization to Cajal bodies	DKC, Hoyeraal-Hreidarsson syndrome, liver disease	Bi-allelic	(170–173)

CST, Ctc1-Stn1-Ten1 complex; DKC, dyskeratosis congenita; TBD, telomere biology disorder.

### 2.2 Functional and cellular consequences of genetic variants implicated in TBD

In all organs, critically short or dysfunctional telomeres lead to cellular senescence or apoptosis (174). In high-turnover tissues such as bone marrow or skin, replicative attrition alone can be sufficient to cause dysfunctional telomeres and lead to disease. In slow-turnover tissues, other acquired hits are thought to be necessary for disease development (175) although the inherited telomere length at birth is crucial in this regard. In the lungs, the key cells affected by telomere dysfunction are the type 2 alveolar epithelial cells, though other cells including lung fibroblasts and immune cells are also influenced by telomere shortening. Critically short telomeres in type 2 alveolar epithelial cells increase susceptibility to exogenous injury, and predispose to a fibrotic response in mouse models (2). In the liver, hepatocyte regenerative capacity is inversely related to telomere length, and it is thought that other hits to the liver contribute to the final development of disease (175, 176).

In general, patients with similar telomere gene mutations may develop different disease manifestations, and patients with similar disease manifestations can carry different mutations. For example, family members with the same telomere gene mutation may display different organ diseases (177). Importantly, individuals carrying TRG variants who inherit shorter telomeres from an affected parent usually develop telomere-related diseases earlier in life, a phenomenon known as genetic anticipation (175). This explains why successive generations in a family develop pulmonary fibrosis at an earlier age. Additionally, successive generations carrying mono-allelic variants that are usually associated with later-onset TBD, can also present with early-onset hematological disease or dyskeratosis congenita (DKC) (175). There is potential for a latency period, where an acquired mutation only leads to disease after several successive generations of carriers (87). A complicating factor is that inheritance of short telomeres can also predispose individuals to disease, even when the individual did not inherit the causative genetic mutation (178).

### 2.3 Strategies for diagnosing TBD

Clinical criteria in combination with leukocyte telomere length, along with screening for genetic mutations, are commonly used for the diagnosis of TBD (179). There are, however, no official guidelines for diagnosing later-onset TBDs, although guidance is provided by the European Respiratory Society statement on familial pulmonary fibrosis (180). Current methods for establishing TBD diagnosis include phenotypic screening, measurement of leukocyte telomere length, and genetic testing for pathogenic mutations in TRGs.

For patients with pulmonary fibrosis, any of the following features qualifies a patient for genetic sequencing:

1. Phenotypic screening for features suggestive of TBD, including.
  - Early greying of hair (<30 years),
  - Otherwise unexplained macrocytosis or cytopenia, myelodysplasia or leukemia,
  - Hepatic abnormalities (cryptogenic elevated liver enzymes, portal hypertension, hepato-pulmonary syndrome, liver cirrhosis),
  - A recognized telomere syndrome such as DKC (characterized by nail dystrophy, oral leukoplakia, abnormal skin pigmentation);
2. Young age at onset of disease (<50 years);
3. Family history of pulmonary fibrosis (in one or more first- or second-degree family members),
4. Identified relative carrying a genetic variant that is pathogenic or likely pathogenic and is known to cause interstitial lung disease (180).

Among potential transplant candidates suffering from other conditions, guidance is much less established and largely relies on personal experience and expert center proposals. For example, among patients with severe emphysematous chronic obstructive pulmonary disease (Global Initiative for Obstructive Lung Disease stage 3 or 4) diagnosed before the age of 65, 1-2% have been found to carry a TRG mutation (181). No recommendation for testing currently exists, and testing for TRG mutations is not routinely performed unless other clinical features suggestive of a short telomere syndrome are present. Similarly, formal guidance on when to screen for and how to diagnose TBDs is lacking for patients with liver disease. Niewisch and colleagues recommend measuring leukocyte telomere length in all patients with unexplained liver disease (including cirrhosis, fibrosis, hepatopulmonary syndrome, and portal hypertension), as well as in patients with a history of alcohol abuse or hepatic infections who also present with other features suggestive of a TBD (182).

Finally, an analogous lack of consensus exists for patients with bone marrow failure or myelodysplastic syndrome. A proposed approach was to evaluate leukocyte telomere length in all patients with a new diagnosis of aplastic anemia, as well as in any patient in whom a TBD is clinically suspected. Genetic testing is then advised if lymphocyte telomere length is below the 10^th^ percentile for age (88).

### 2.4 Measurement of leukocyte telomere length

Very short leukocyte telomere length can aid in the diagnosis of a TBD in patients without a known genetic mutation, those carrying a previously undescribed mutation, variants of uncertain significance, or in cases of suspected genetic anticipation. A telomere length below the first percentile for age is highly suggestive of a telomere-related disease, although not all patients with TBDs exhibit this finding. Leukocyte telomere length between the 1^st^ and 10^th^ percentiles for age may also support the diagnosis in the appropriate clinical context but can be observed in individuals without telomere-related disease (4). Importantly, a normal leukocyte telomere length does not necessarily exclude TBD.

Several techniques are available for measuring leukocyte telomere length, including flow cytometry with fluorescence *in situ* hybridization (FlowFISH), quantitative polymerase chain reaction (qPCR), next-generation sequencing, and Southern blotting. FlowFISH is considered the clinical gold standard for diagnostic use, due to its high inter-laboratory reproducibility and the availability of nomograms that are consistent across populations. However, it requires fresh blood samples, specialized equipment, and technical expertise, which limits its accessibility. By contrast, qPCR is more widely accessible because it does not require specialized instrumentation, but it demonstrates lower reproducibility across laboratories (183, 184).

## 3 Mechanistic links between TBD and hematologic diseases in the setting of solid organ transplant

### 3.1 Links between TBD, bone marrow failure and altered immunity

#### 3.1.1 TBD and bone marrow failure

The bone marrow is one of the highest turnover compartments of the body, meaning that hematopoietic stem cells (HSC) are under constant replicative and selective pressure. Unsurprisingly, telomerase activity can be detected in HSC and normal telomere length supports their proliferative potential, even though telomeres naturally shorten as they proliferate, even in this cellular population (185, 186). Based on experimental murine evidence, HSC with short telomeres accumulate DNA damage at the quiescent stage, which triggers the initiation of apoptosis and senescence pathways at the transition from quiescent to active HSC (90). The activation of p53 and p21 results in impaired proliferation capacity, increasing the replicative burden on the effective survivor population, leading to increased cell divisions and telomere attrition, culminating in stem cell exhaustion (187, 188). In humans, the bone marrow of (pediatric) TBD patients displays signs of p53 activation and apoptosis (188) and bone marrow aspirates of DKC patients have decreased cellularity, pointing toward similar processes. Consequently, patients with TBD are at risk of developing bone marrow failure, particularly within the DKC population, where it affects 80-90% of individuals by age 30 and represents a leading cause of death (189). Nonetheless, even adults with later-onset symptoms are at risk. In one cohort, 70% of adult TBD patients without clinical features of DKC displayed stigmata of bone marrow failure, most frequently in the form of leukopenia, thrombocytopenia and anemia, alongside bone marrow hypoplasia or aplasia (190). In addition, 40% of a selected population of patients presenting with pulmonary fibrosis and short telomeres were found to have a hypocellular bone marrow (191). Further highlighting the impact of short telomeres, 30% of patients with aplastic anemia have telomere length below the 1^st^ percentile (14), and approximately 5-10% carry a mutation in a TRG (89, 192). Overall, patients with TBD are therefore at increased risk of bone marrow exhaustion and related hematologic diseases.

#### 3.1.2 TBDs are associated with immunologic alterations

Multiple immunologic alterations may arise in patients with TBD, with lower T-cells counts manifesting in 45% of cases (193, 194), but also B-cell lymphopenia or neutropenia (193, 195). T-cell lymphopenia is characterized by depletion of naïve CD4^+^ and CD8^+^ T-cells alongside a partially decreased thymic output mirroring the T-cell changes witnessed in older adults with normal telomere length (193–195). Similarly to aging, these lymphocytes also display augmented signs of apoptosis, albeit they show a disease-specific upregulation of p53, as could be expected from the signal elicited by short telomeres (194). In a more recent report, T-cell lymphopenia was absent in a subset of patients with short telomeres, hinting at stochastic processes or additional factors. In fact, HSC from mice with short telomeres do not show signs of apoptosis, but present a skewed differentiation toward megakaryocytic/myeloid lineage differentiation with impaired self-renewal, which is also present in *TERT/TERC* mutated patients and may (partly) drive T-cell lymphopenia (196). In theory, impaired DNA damage tolerance mechanisms could additionally be involved in these observations, as mice deficient for DNA damage tolerance similarly present with a decrease in lymphoid progenitors and HSC fitness, reminiscent of premature aging (197). Finally, T-cells in TBD patients show not only signs of aging, but also markers of activation and exhaustion, underlining the complex immunological environment present in these patients (195).

Recent screening of 419 patients with common variable immune deficiency identified a variant in a TRG in 1% of the cohort (198), suggesting that B-cells may also be severely affected by TRG mutations. Although B-cell composition is less frequently altered in TBD patients, an overall reduction in B-cell numbers is observed, alongside a reduction of the unswitched and IgM^+^ B-cell pools. These immature B-cells show signs of apoptosis, with an increased proportion of CD95(Fas)^+^CD19^+^ cells (195). Similarly, TBD patients also show decreases in IgM levels and, to a smaller extent diminished IgG and IgA levels (194). The extent to which these humoral immunity changes affect the clinical phenotype of patients is currently unknown. While TBD patients are at increased risk for opportunistic infections, the reported infections mainly include varicella zoster reactivation, severe cytomegalovirus infection, and *Pneumocystis jirovecii* pneumonia, which are more indicative of T-cell immunodeficiency rather than B-cell dysfunction (194).

### 3.2 TBD carriers are at increased risk for malignancy

#### 3.2.1 Links between short telomeres and cancer

Critically shortened telomeres can trigger a DNA damage response, resulting in apoptosis or senescence, two key mechanisms of tumor suppression (199, 200). Consequently, telomeres can act as protective mechanisms against oncogenesis by restricting the replication of precancerous cells. In line with this, experimental *in vitro and in vivo* models, in which telomere function was altered, display impaired tumor cell growth as well as signs of apoptosis and senescence (199, 201, 202). Dysfunctional telomere sensing results in the activation of p53 and p16 signaling (203–206), which acts as key components of these oncogenic safeguards. Indeed, short telomeres require p53 to exert a protective effect in several murine cancer models and loss of p53 in combination with short telomeres results in shortened tumor latency (207). In addition, tumors derived from late-generation Myc mTR-/- mice (deficient in the RNA component of telomerase), present with a loss of this protein in this model of Burkitt lymphoma with short telomeres due to mouse telomerase RNA deficiency (199). When the p53 or Rb tumor suppressors pathways become dysfunctional, cells can bypass this proliferative barrier, leading to further cell division, extensive telomere shortening and uncapping with the activation of DNA damage response (208–210). During this telomeric crisis, end-to-end fusions between chromosomes or non-telomeric double strand breaks can occur, giving rise to dicentric chromosomes and genomic instability (207, 209, 211). These chromosomal anomalies will trigger cell death through autophagy and innate immune pathways (210, 212), thereby serving as a second barrier to malignant transformation. However, the chromosomal instability generated during crisis may also provide a substrate for oncogenic progression. Indeed, cancer associated mechanisms, including translocations, amplifications, loss of heterozygosity and chromothripsis (a shattering of a chromosomal region, followed by random rejoining) can be found during this phase (207, 208). As a consequence, cells that escape death after telomere crisis, either because of the restoration of telomerase activity or (undocumented) alteration of autophagy may progress toward oncogenesis (213). Supporting this model, in a majority of cancers telomere shortening is present as compared to related healthy tissue (214), and telomerase activity is largely detectable alongside *TERT* reactivation and alternative lengthening of telomeres mechanisms (214).

#### 3.2.2 Cancer in TBD patients

In patients with TBDs, cancer frequency is heightened but remains a relatively rare entity, affecting 10-15% of individuals (215–217). Myelodysplastic neoplasms (MDS), acute myeloid leukemia (AML) and oral squamous cancers are the most prevalent in this population with an up to 145-times higher frequency than would be expected in a sex, race and gender-matched population (218). DKC patients seem to be at particular risk for squamous cell cancers (216, 218), as they are overrepresented in individuals developing these malignancies (219). Notably, whole genome sequencing of squamous cell cancers from patients with short telomeres revealed the overwhelming presence of *TP53* loss-of-function alterations, shortened tumor telomere length and activation of telomere maintenance mechanisms (217). Evaluation of telomere-driven genomic instability did not show signs of chromothripsis or translocations surrounded by telomere repeats (217), suggesting the involvement of other mechanisms. Importantly, T-cell deficiency has been linked with the development of solid cancer (mostly squamous cell cancers) in patients (193), while implantation of immunogenic tumor cells in late-generation mTR^-/-^ mice, which mirror this T-cell lymphopenia, results in tumor implantation and development, in contrast to early-generation or wild-type T-cell competent animals (217).

##### 3.2.2.1 Myelodysplastic neoplasms

MDS constitute a heterogeneous group of clonal HSC disorders characterized by ineffective hematopoiesis and a high risk of progression toward AML, with dysfunctional HSC and progenitor cells playing an important role (220–222). MDS patients display significantly shorter telomere lengths compared to healthy controls (223), and short telomeres are a negative prognostic marker in the disease (224, 225), suggesting potential links with TBD. As a matter of fact, 2.7% of MDS patients without prior suspicion for TBD harbor rare variants in *TERT* (226). Congruently, MDS is the most frequent oncologic manifestation in TBD patients, representing 60% of malignancies in this population, and occurs on average 20 years earlier than in the general population. Prognosis remains poor even though mortality is also related to extra-hematopoietic manifestations of TBD (218).

Bone marrow aspirates from these patients show signs of hypoplasia in 50% of cases, in contrast to the 10-20% observed in the general MDS population. Patients are typically diagnosed three decades later than those with aplastic anemia, suggesting an adult TBD-associated permissive telomeric environment that allows oncogenic drift over time (218, 227). Nonetheless, cytogenetic analysis do not reveal major differences compared to unselected MDS patients; for example, rates of monosomy 7 are similar (218), and somatic reversion mechanisms fueling malignancy remain rare (228), hinting that additional drivers contribute to disease pathogenesis. Experimental data from a TERT murine model show that telomere dysfunction and DNA damage can lead to aberrant splicing, hindering progenitor differentiation and leading to decreased levels of key factors such as DNMT3A, a DNA methyltransferase associated with hematologic malignancies, thereby promoting epigenetic changes that may further drive malignancy (229).

##### 3.2.2.2 Acute myeloid leukemia

AML can arise primarily, or as a secondary event following MDS, complicating 5-35% of cases depending on risk group stratification (230). AML originates from uncontrolled proliferation of clonal hematopoietic cells and carries a dismal prognosis, with a 5-year overall survival of approximately 30% (231). Patients with AML have significantly shorter telomeres compared to healthy controls (232), and a study of an unselected AML cohort identified TERT variants with reduced enzymatic function in 8% of patients (233). In addition, cytogenetic analysis of these patients revealed karyotypic anomalies in all of them, with higher rates of inversion 16 and trisomy 8 than expected (233), even though this was not confirmed in a later well-characterized TBD cohort (218). Finally, patients with TBDs represent a high-risk group for AML, with an observed frequency approximately 20 times higher than expected, and AML accounting for 17% of all cancers in this population (218).

**Figure 1:**
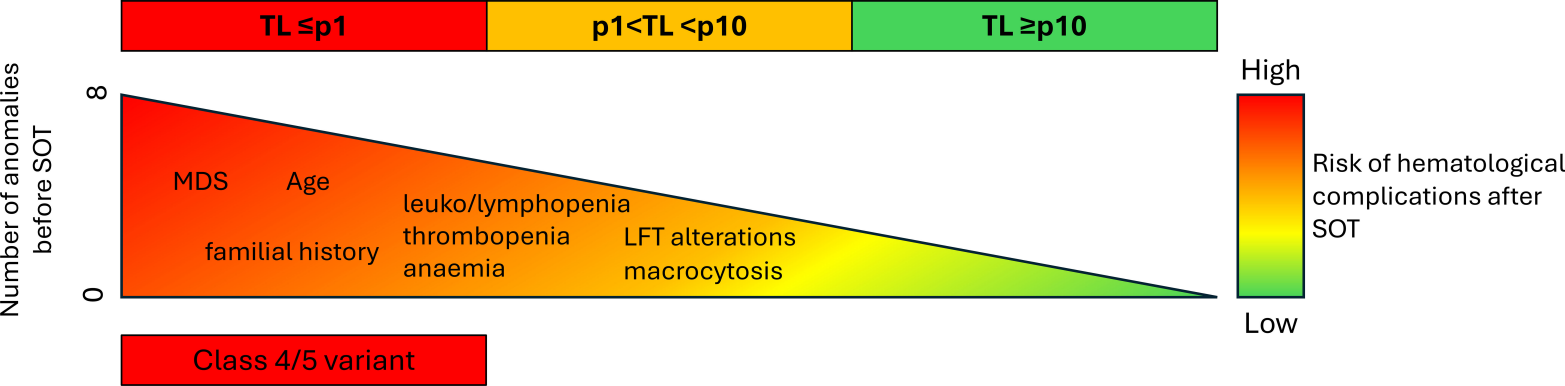
Schematical risk model for hematological complications following solid organ transplantation (SOT) based on current knowledge.

Overall, patients with TBDs are at increased risk for hematologic disease, with presentations influenced predominantly by age and telomere length. Very short telomeres lead to stem cell proliferative failure and aplasia, whereas longer telomeres permit limited replication, potentially facilitating oncogenic drift and the subsequent development of MDS and AML. Additional factors can also influence hematological risk and are probably cumulative. **Figure 1** schematizes the relative burden of shortened telomere length, the presence of pathogenic variants in telomere maintenance genes, as well as the importance of cumulative factors (e.g., MDS, age, familial history, cytopenia) in determining the risk of hematological complications after solid organ transplant.

## 4 Implications of telomere biology disorders in solid organ transplantation

The relevance of TBDs, including both short telomeres and genetic variants in telomere maintenance-related genes, to outcomes following solid organ transplantation remains largely unexplored, except in lung transplantation. In this field, the literature has expanded steadily over the last decade, though it has mostly focused on patients with interstitial lung disease (ILD).

### 4.1 Telomere biology disorders in lung transplantation

TBDs have emerged as clinically relevant in lung transplantation, particularly among candidates with ILD. Patients with TBD are overrepresented among lung transplant candidates, and pathogenic or likely pathogenic variants in telomere maintenance genes have been reported in up to one quarter of lung transplant candidates with idiopathic pulmonary fibrosis (IPF) (234). Severe emphysema has also been linked to TBD, broadening the spectrum of telomere-related lung diseases (181). However, the clinical impact of TBD in the context of lung transplantation for COPD remains unknown.

The first observational cohorts of lung transplant recipients with TBD unequivocally highlighted the high rate of hematological complications induced by immunosuppression. Silhan et al. reported a seminal series of 8 TBD patients transplanted for IPF, 7 of whom carried *TERT* or *TERC* variants. All experienced hematological complications, particularly transfusion-requiring thrombocytopenia and leukopenia necessitating immunosuppressive regimen reduction to two drugs instead of the standard three-drug maintenance therapy. Frequent renal failure and prolonged hospital stays were also noted (235). Similarly, Borie et al. described 9 IPF patients with *TERT* or *TERC* variants who displayed high rates of myelodysplastic syndrome (MDS), anemia, and neutropenia post-transplant. Notably, these complications were more common in patients with pre-transplant thrombocytopenia or MDS, suggesting limited baseline bone marrow reserve (236). These findings were reinforced in another cohort of 14 ILD patients with *TERT* or *TERC* variants: 12 developed leukopenia, all 14 anemia, and 5 developed thrombocytopenia (15).

Larger studies focusing on age-adjusted telomere length have since provided further, though sometimes conflicting, evidence. In a cohort of 82 ILD lung transplant recipients, those with short telomeres (n=26, <10^th^ percentile) showed no difference in hematological complications compared to controls (n=56), but had reduced chronic lung allograft dysfunction (CLAD)-free survival, higher mortality, and more frequent grade 3 primary graft dysfunction (237). Conversely, in a cohort of 175 lung transplant recipients with COPD, ILD, pulmonary arterial hypertension or cystic fibrosis, those in the lowest telomere length quintile had higher rates of leukopenia, but not thrombocytopenia or anemia (238). This was partially supported by another study of 69 real-world lung transplant recipients (including ILD, COPD, and cystic fibrosis patients). Recipients in the lowest tertile of telomere length were more likely to develop GM-CSF-requiring leukopenia. Interestingly, post-transplant telomere shortening rate was explored, but not associated with outcomes (239). In a comparative study of 262 lung transplant recipients, patients with variants in *TERT*, *RTEL1*, or *PARN* (n=31) were more likely to develop anemia than non-variant carriers (n=231), but not leukopenia or thrombocytopenia, again supporting the concept of impaired bone marrow reserve in lung transplant recipients with TBD (240). Similarly, Hannan et al. compared 72 IPF and 72 matched non-IPF lung transplant recipients, showing that patients with short telomeres (≤ 10^th^ percentile) were more likely to require immunosuppressive tapering due to cytopenia, as well as bone marrow biopsy for dysfunction (241). A recent genetic study further emphasized the hematological burden of TBD in lung transplant recipients. All 38 ILD lung transplant recipients with *TERT*, *TERC*, or *RTEL1* variants developed anemia; two-thirds developed thrombocytopenia, and one-third developed neutropenia requiring GM-CSF or immunosuppressive reduction (242).

While the evidence consistently indicates increased bone marrow insufficiency in TBD lung transplant recipients – likely due to combined exposure to intensive immunosuppression and myelotoxic anti-infective agents such as valganciclovir and trimethoprim-sulfamethoxazole – data remain limited regarding other hematologic complications such as MDS, post-transplant lymphoproliferative disorders (PTLD) or thrombotic microangiopathy (TMA). Borie et al. reported a high incidence of MDS post-transplant (50%), but this largely occurred in patients with pre-existing bone marrow abnormalities (236). Subsequent studies have not confirmed this association. With regard to PTLD, IPF has recently been recognized as a risk factor for EBV-mediated PTLD after lung transplant (243). Given that impaired T-cell immunity in TBD patients reduces CMV control after transplantation (244), one could hypothesize similar mechanisms may impair EBV control, potentially predisposing to PTLD, although this has not been mechanistically proven. Finally, although TMA cases have been reported in lung transplant recipients with telomere maintenance gene mutations (236, 242), sometimes with fatal outcomes, the scarcity of reports prevents firm conclusions.

Taken together, these findings suggest that TBD are consistently associated with hematological complications after transplantation, particularly in ILD recipients. Associations with immunological outcomes such as acute rejection and CLAD have also been reported, though inconsistently, likely reflecting heterogeneous study designs, patient populations, and telomere measurement methods (245). Importantly, TBD are not considered an absolute contraindication to transplantation, but their recognition has major implications for risk stratification, peri-transplant counseling, and individualized immunosuppressive management (246, 247). Emerging data also suggest that *donor* telomere length may influence lung transplant outcomes (238), further supporting the need to integrate telomere biology into future prospective studies.

Overall, the literature published over the past decade is marked by substantial heterogeneity, limiting the ability of clinicians to draw firm conclusions regarding the true impact of TBD on hematologic risk after lung transplantation. Considerable variability exists in the methods used to assess telomere length, ranging from Southern blot to qPCR and flow-FISH, as well as in the reference populations and normative datasets applied. Correspondingly, the thresholds used to define “short” telomeres differ widely across studies. Moreover, some reports center exclusively on telomere length, whereas others investigate pathogenic variants in telomere-related genes, and only a minority integrate both approaches within the same cohort. Finally, most available data derive from small, retrospective, single-center series, with a notable absence of prospective or multicenter studies. **Table 2** summarizes these methodological differences across published cohorts and underscores the need for greater standardization in future research, including (1) harmonization of telomere length measurement techniques, (2) consistent and assay-appropriate telomere length cut-offs, (3) combined assessment of telomere length and telomere-related gene variants to improve interpretability and reproducibility and (4) implementation of prospective, multicenter, ideally international studies to generate robust, generalizable evidence.

**Table 2:** Comparative overview of published cohorts, case series, and case reports describing hematological complications following solid organ transplantation in patients with telomere biology disorders.

Organ(s) involved	Study population	Organ disease (n patients)	Age at Tx*	Gender (F/M)	Countries	Inclusion period	Mutations reported (n patients)	TL measurement technique	TL cut-off	Study design	Ref.
Lung	8 LuTR with TBD	IPF	52	4/4	USA, Australia, Sweden	2004-2013	TERT (5) TERC (2)	FlowFISH	<1^st^ percentile	Retrospective cohort	(235)
Lung	9 LuTR with TBD	Pulmonary fibrosis	50	2/7	France	2009-2013	TERT (6) TERC (3)	Southern blot	Comparison to age-matched controls	Retrospective cohort	(236)
Lung	14 LuTR with TBD	UIP (8) Other ILD (6)	60.5	5/9	USA, Australia	2005-2014	TERT (10) TERC (1)	NA		Retrospective cohort	(15)
Lung	82 LuTR with ILD: - 26 with short TL - 56 with normal TL	IPF (50) CTD-ILD (10) HSP (8) Other (14)	59 (mean)	25/57	USA	2007-2014	NA	qPCR	<10^th^ percentile	Prospective observational cohort	(237)
Lung	175 LuTR	COPD (43) PAH (11) CF (21) ILD (100)	54 (mean)	83/92	USA	2000-2015	NA	qPCR	Lowest quintile of study population	Retrospective cohort	(238)
Lung	69 LuTR (56 with TL)	ILD (32) COPD (18) CF (13) Other (6)	57.9	23/46	USA	2014-2018	NA	qPCR *(! measured after LTx)*	Lowest tertile of study population	Prospective observational cohort	(239)
Lung	262 LuTR - 31 with TBD - 231 without TBD	IPF (213), CTD-ILD (30), NSIP (19)	65	58/204	USA	NR	TERT RTEL1 PARN	NA		Retrospective cohort	(240)
Lung	144 LuTR - 72 with IPF - 72 non IPF	IPF (72) COPD (50) Other (22)	65	IPF: 16/56 Non-IPF: 34/38	USA	2015-2019	TERT (2) TERC(1) RTEL1 (6) PARN (6) TINF2 (1) NAF1 (1) DKC1 (2)	FlowFISH and TelSeq assay	≤10^th^ percentile	Retrospective cohort	(241)
Lung	38 LuTR with TBD	IPF (27) Unclassifiable ILD (4) Other ILD (7)	53.6	12/26	France, Belgium, Switzerland	NR	TERT (23) TERC (9) RTEL1 (6)	qPCR	NR	Retrospective cohort	(242)
Liver	40 advanced hepatic disease with TBD - 20 LiTR - 20 no LiTR	Cirrhosis (9) HPS (14)	29	12/28	USA, Germany, Italy, Brazil	NR	TERT (12) TERC (1) RTEL 1 (2) DKC1 (3) PARN (2) TINF2 (9) CTC1 (1)	NR	Low: <10^th^ percentile, Very low: <1^st^ percentile	Retrospective cohort	(248)
Liver	2 LiTR with TBD	HPS (2)	23, 29	1/1	USA	NR	TERT (1)	NR	<1^st^ percentile	Case series	(249)
Liver, Lung	1 sequential Li/LuTx	Cryptogenic cirrhosis, IPF	57	0/1	USA	NR	NA	FlowFISH	<5^th^ percentile	Case report	(250)
Liver	1 LiTx	DKC, cryptogenic cirrhosis	34	0/1	India	2013	NA	NA		Case report	(251)
Liver, Lung	1 sequential Li/LuTx, 1 combined Li/LuTx	HPS, IPF	35, 52	0/2	Belgium	2015	TERT (1), TERC (1)	NA		Case series	(252)
Liver, Lung	1 combined Li/LuTx	HPS, IPF	52	0/1	Switzerland	2017	NA	FlowFISH	<1^st^ percentile	Case report	(253)
Liver, Lung	4 TBD patients, 1 underwent sequential Li/LuTR	HPS, IPF	35	0/1	Netherlands	NR	POT1	qPCR	<10^th^ percentile	Case series	(127)
Liver, Kidney	1 combined K/LiTx	ESRD, congenital hepatic fibrosis	22	0/1	India	NR	TINF2	NA		Case report	(254)
Liver	1 LiTx	HPS	11	0/1	USA	NR	DKC1	FlowFISH	<1^st^ percentile	Case report	(33)
Liver, lung	1 combined Li/LuTx	HPS	5	0/1	Corea	NR	TINF2	NA		Case report	(255)
Liver	1 LiTx	HPS associated to Coats plus syndrome	16	0/1	Japan	NR	NR	NA		Case report	(256)

COPD, chronic obstructive pulmonary disease; CTD, connective tissue disease; DKC, dyskeratosis congenita; ESRD, end-stage renal disease; ILD,. interstitial lung disease; IPF, idiopathic pulmonary fibrosis; HPS, hepatopulmonary syndrome; K, kidney; LiTR, liver transplant recipients; LuTR, lung transplant recipients; NA, not assessed; NR, not reported; TBD, telomere biology disorder; TL, telomere length; Tx, Transplantation; UIP, usual interstitial pneumonia.

* Reported ages represent median ages for cohort studies, except where mentioned otherwise.

### 4.2 Telomere biology disorders in non-lung solid organ transplantation

In contrast to lung transplantation, evidence on TBD-related hematological complications after other solid organ transplants (kidney, liver, heart) is extremely limited and mostly restricted to case reports and small series. This is likely due to both the less established role of TBDs in the underlying diseases of these organs and the lower enrichment of TBDs among heart and kidney transplant candidates.

Notably, a recent cohort study retrospectively reported the outcomes of 83 patients with TBD-related hepatic disease (i.e., cirrhosis and hepatopulmonary syndrome), with TBD-related defined as short telomeres (<10^th^ percentile), pathogenic variant of telomere maintenance-related genes and/or clinical presentation (248). Among them, liver transplantation was performed in 20 patients (5 of which were already published in two previous case series/reports (33, 257). The authors observed improved survival as compared to non-transplanted patients. In some patients, anemia and bone marrow cellularity improved post-transplant, possibly due to the resolution of cirrhosis-related gastrointestinal bleeding, while one patient developed aplastic anemia. No hematological malignancy was reported.

Several small case series and individual reports have described liver transplantation in patients with TBDs. Oseini et al. reported two patients – one with nodular regenerative hyperplasia and a TERT mutation, and one with DKC without a reported mutation – who underwent liver transplantation for hepatopulmonary syndrome several years after bone marrow transplantation. Both patients had relatively uneventful post-transplant courses under standard immunosuppression and demonstrated progressive resolution of hypoxia (249). In another report, a patient with short telomere length (<5th percentile for age, no genetic testing performed) underwent liver transplantation for cryptogenic cirrhosis and later developed pulmonary fibrosis requiring single-lung transplantation; no specific complications were reported 18 months post-transplant (250). Finally, a living-donor lobar liver transplantation was performed in a patient with DKC (no mutation reported) who had liver cirrhosis and hepatopulmonary syndrome. As in the previous case, no specific complications were observed during 22 months of follow-up, and hypoxia resolved despite preexisting ILD lesions (251). Of note, in TBD patients undergoing liver transplantation, pulmonary disease beyond hepatopulmonary syndrome has been reported. For example, an early report described two patients – one with a *TERT* mutation and one with an *RTEL1* mutation – who underwent liver transplantation for hepatopulmonary syndrome and subsequently developed progressive pulmonary fibrosis. In one case, this occurred within 18 months of transplantation, while in the other it developed 12 years later (258). Although not specifically focused on hematologic complications after organ transplantation, these reports nevertheless suggest that such procedures are feasible in patients with TBDs.

Other reports involve multi-organ transplantation. Two patients with *TERT* or *TERC* variants underwent combined liver-lung transplantation (one sequential, one simultaneous) with divergent hematological outcomes: either improvement or worsening of pre-existing thrombocytopenia, the latter requiring repeated transfusions. These discrepancies may reflect differences in transplant timing (252). In another report, a patient with very short telomere length (<1st percentile for age, no mutation identified) successfully underwent combined lung and liver transplantation for pulmonary fibrosis and hepatopulmonary syndrome. This patient had pancytopenia prior to transplantation, which normalized following the procedure, although after receiving danazol therapy (253). Another report described a patient with a *POT1* mutation who underwent liver transplantation for cirrhosis, followed two years later by lung transplantation for pulmonary fibrosis. Although the patient had a prior history of anemia and thrombocytopenia, both had resolved before the liver transplant. No hematologic complications occurred under standard immunosuppression; however, the patient experienced recurrent fungal and bacterial infections (127). Although kidney transplantation is not commonly reported in patients with TBD, a case report describes a combined liver-kidney transplant in a patient with a mutation in *TINF2*, which was complicated by postoperative pancytopenia and bone marrow hypocellularity, with thrombocytopenia persisting at 9 months post-transplant (254).

More specifically, there are also several reports of successful liver transplantation in children with DKC. In one case, suspected PTLD developed but resolved following reduction of immunosuppression, and no additional infectious or hematologic complications were reported during 10 years of follow-up (33). Another pediatric patient with a *TINF2* mutation underwent combined liver-lung transplantation for hepatopulmonary syndrome two years after bone marrow transplantation. Outcomes were favorable 11 months post-transplant, with no specific hematologic or infectious complications reported under standard immunosuppression (255). Finally, a patient with Coats plus syndrome who developed hepatopulmonary syndrome underwent living-lobar liver transplantation, which successfully resolved hypoxia, and the patient remained well at one-year follow-up (256).

Overall, the evidence regarding TBD-related complications in liver (or other non-lung solid organ) transplantation is even more limited and heterogeneous than in lung transplantation, consisting almost entirely of isolated case reports and small case series, with the exception of a single larger retrospective cohort. This scarcity of systematically collected data makes it difficult to assess true hematologic risk, to distinguish TBD-related manifestations from organ-specific complications, or to establish standardized management strategies. **Table 2** summarizes the available reports and highlights the variability in patient populations, genetic characterization, and telomere length measurement techniques, underscoring the need for more consistent data collection and prospective multicenter studies in this setting.

To date, no studies have systematically evaluated hematological complications of TBDs in isolated heart or kidney transplantation. The scarcity of data likely reflects the fact that TBD-related diseases rarely affect these organs directly. This lack of evidence prevents drawing any conclusions regarding the eligibility of TBD candidates for such procedures or formulating recommendations on post-transplant immunosuppressive management in this subset of patients.

## 5 Screening and mitigation strategies in TBDs

Given the scarcity of systematic data, optimal screening and management of TBD patients prior to solid organ transplantation remain undefined, although emerging recent literature arises in the field (259). **Table 3** provides a structured overview of practical considerations for pre-transplant evaluation, risk stratification, and post-transplant monitoring in this patient population. These recommendations are primarily based on the clinical experience of the authors and limited reports from other centers and are intended to highlight potential areas of concern, rather than to serve as formal guidelines.

**Table 3:** Considerations for screening for TBD, as well as pre- and post-transplant management in lung and liver transplant candidates with a TBD.

Clinical context	Lung transplantation	Liver transplantation
Whom and how to evaluate for TBD prior to transplantation	Genetic testing in patients with pulmonary fibrosis if: • Other clinical features suggestive of a TBD^#^ • Family history of pulmonary fibrosis in 1^st^ or 2^nd^ degree relative • Disease onset prior to age 50 years • Family history suggestive of a TBD* • Relative carrying a genetic telomere-related variant that is pathogenic	Genetic testing in patients with liver cirrhosis or HPS if: • Other clinical features suggestive of a TBD^#^ • Family history suggestive of a TBD* • Relative carrying a genetic telomere-related variant that is pathogenic
Pre-transplantation evaluations in patients with (suspected) TBD	• Hematology consult, bone marrow biopsy in case of unexplained cytopenia • Liver ultrasound, fibroscan, hepatology consult of abnormal • Saline-agitated echocardiogram if abnormally low diffusion capacity or known liver disease	• Hematology consult, bone marrow biopsy in case of unexplained cytopenia • Saline-agitated echocardiogram to assess hepatopulmonary syndrome • Spirometry, diffusion capacity, HRCT-chest, pulmonology consultation if abnormal
Post-transplantation considerations in patients with (suspected) TBD	• Basiliximab favored as induction therapy • MMF favored over azathioprine as maintenance therapy • Reactive tapering of immunosuppressive therapy in case of cytopenia, CMV viremia, or clinically relevant immunodeficiency • Consider life-long prophylaxis in CMV-negative recipients with CMV-positive donors, preference for letermovir over valganciclovir • Consider atovaquone instead of trimethoprim-sulfamethoxazole in case of refractory cytopenia	• Basiliximab favored as induction therapy • MMF favored over azathioprine as maintenance therapy • Reactive tapering of immunosuppressive therapy in case of cytopenia, CMV viremia, or clinically relevant immunodeficiency • Consider life-long prophylaxis in CMV-negative recipients with CMV-positive donors, preference for letermovir over valganciclovir • Consider atovaquone instead of trimethoprim-sulfamethoxazole in case of refractory cytopenia • Yearly monitoring for lung disease with pulmonary function testing and HRCT-scan of the chest

^#^ Clinical features suggestive of a TBD include early greying of hair (<30 years), otherwise unexplained macrocytosis or cytopenia, myelodysplasia or leukemia, hepatic abnormalities (cryptogenic elevated liver enzymes, portal hypertension, hepato-pulmonary syndrome, liver cirrhosis), pulmonary fibrosis, or a recognized telomere syndrome such as DKC (nail dystrophy, oral leukoplakia, abnormal skin pigmentation).

* Family history suggestive of a TBD includes liver disease, hematological disease, DKC, and pulmonary fibrosis.

CMV, cytomegalovirus; HPS, hepatopulmonary syndrome; HRCT, high-resolution computed tomography; MMF, mycophenolate mofetil; TBD, telomere biology disorder.

Telomere length measurement and genetic screening for TBDs should be offered to lung transplant candidates who display clinical features suggestive of such disorders (180). A similar approach may be expanded to patients with severe emphysema, although supporting data remain very limited. For liver transplant candidates, we likewise recommend this strategy. A TBD can be confirmed by the identification of a known pathogenic mutation. In the absence of such a mutation, low leukocyte telomere length (<10^th^ percentile for age, and particularly <1^st^ percentile for age) in the appropriate clinical context can also support the diagnosis of TBD. The management suggestions outlined below are specific to patients with confirmed TBD, but may be considered in patients with suspected TBD, in whom a high index of suspicion should be maintained.

Pre-transplant assessment of both lung and liver transplant candidates with a known or suspected TBD should include hematology consultation, with bone marrow biopsy considered in the presence of unexplained blood count abnormalities (246). This evaluation is more challenging in liver transplant candidates, where splenomegaly secondary to portal hypertension can also cause anemia and thrombocytopenia. In patients with established bone marrow disease related to TBD, hematology input is essential to assess post-transplant risk of cytopenia and hematological malignancy. In rare cases, bone marrow transplantation may be considered prior to or after solid organ transplantation.

In lung transplant candidates with a TBD, pre-transplant evaluation should include liver enzymes testing and liver imaging, including elastography (e.g., Fibroscan), with abnormal findings warranting hepatology consultation. Candidates with unexpectedly low diffusion capacity for their degree of pulmonary disease, or with established liver disease, should also be evaluated for hepatopulmonary syndrome using saline-agitated echocardiography. In the case of severe liver disease, referral to a hepatologist is recommended, and combined or sequential lung-liver transplantation should be considered.

For liver transplant candidates with a TBD, pre-transplant evaluation should include standard screening for hepatopulmonary syndrome. Pulmonary assessment in these candidates should also encompass pulmonary function tests, including diffusion capacity, as well as high-resolution computed tomography of the chest to detect interstitial lung abnormalities or ILD. If abnormalities are identified, consultation with a transplant pulmonologist is recommended to determine their relevance to the proposed liver transplant and to assess whether combined or sequential lung-liver transplant should be considered.

For organ transplant candidates with a TBD who are CMV-seronegative, an organ from a CMV-negative donor is preferred. However, this is often difficult to achieve due to the general shortage of donor organs, and we, in line with Courtwright and colleagues, do not recommend delaying lung transplantation to wait for a CMV-negative donor (247).

Post-transplantation immunosuppressive therapy for both lung and liver transplant recipients can be tailored for TBD patients. Induction therapy with basiliximab should be preferred over other agents, as they may induce prolonged T-cell depletion. For maintenance therapy, mycophenolate mofetil is preferred over azathioprine, which is more commonly associated with bone marrow suppression. Experts in the field have suggested upfront modification of immunosuppression in lung transplant recipients with TBD and short telomere length (246). However, we favor a reactive approach, adjusting immunosuppressive therapy in response to cytopenia or CMV viremia, since these complications are generally manageable, and many of these patients tolerate standard immunosuppressive treatment well. This approach may, in turn, be associated with a lower risk of CLAD, although this hypothesis remains speculative.

For persistent cytopenia, consultation with hematology should be sought, and a bone marrow transplant can be considered in selected patients. There is not enough evidence to recommend the use of danazol for bone marrow disease after solid organ transplantation in patients with TBD (260).

Antimicrobial agents with myelosuppressive potential should be avoided whenever possible. Prophylactic trimethoprim-sulfamethoxazole to prevent *Pneumocystis jirovecii* pneumonia can be substituted with atovaquone or aerosolized pentamidine, as suggested by some authors (246, 261). In our experience, however, low-dose trimethoprim-sulfamethoxazole is usually well-tolerated and can be proposed as first-line prophylaxis, with a potential switch to atovaquone or pentamidine if hematologic complications arise.

Determining the optimal strategy for CMV prophylaxis is more challenging. In particular, for mismatched recipients (CMV-seronegative recipients receiving an organ from a CMV-positive donor), lifelong CMV-prophylaxis should be considered (247). Valganciclovir is the most commonly used agent, but it carries a high risk of myelosuppression in both lung and liver transplant recipients (262). Alternative agents without myelosuppressive effects, such as letermovir, are preferred; however, their use is currently limited by higher costs (263). In our experience, reducing immunosuppression, switching cell cycle inhibitors to everolimus, or using CMV-specific intravenous immunoglobulins can be considered as treatment options for transplant recipients with TBD who develop refractory CMV viremia or cannot tolerate antiviral therapy.

In lung transplant recipients without evidence of liver disease prior to transplantation, we do not recommend routine liver-specific monitoring beyond standard liver function tests during regular follow-up visits, as the prevalence of clinically significant liver disease in these patients appears lower than in the reverse scenario. In contrast, liver transplant recipients with a TBD frequently have lung involvement, with lung disease present in up to 40% of patients (248). Therefore, we recommend annual monitoring with pulmonary function testing and high-resolution computed tomography of the chest, even in the absence of pre-transplant lung disease. For patients with pre-existing lung disease, more frequent pulmonology evaluations are warranted to enable timely initiation of antifibrotic therapy and consideration for potential lung transplantation.

## 6 Conclusions and future perspectives

Although large studies specifically designed to address hematologic complications after solid organ transplantation in patients with TBDs are still lacking, the literature – particularly in lung transplantation – is steadily expanding, yielding intriguing but sometimes conflicting results. Three major limitations of the current data should be considered before summarizing any recommendations. First, the definition of “TBD” varies widely across studies, with terms such as “short telomere syndromes,” “telomeropathies,” and “TBD” often used interchangeably. Thresholds for defining short telomeres also differ, ranging from below the 1^st^ percentile to <10^th^ percentile, or even the lowest quartile or tertile of study populations. This variability complicates direct comparisons, further compounded by differences in telomere length measurement techniques, as qPCR and FlowFISH offer distinct reproducibility and inter-laboratory consistency profiles. Moreover, while initial studies focused primarily on *TERT* and *TERC*, subsequent work has expanded to include a growing number of TRGs, suggesting that earlier reports may have underrepresented the true prevalence of pathogenic variants due to methodological limitations. Second, the largest and most robust studies have been conducted in the setting of lung transplantation, almost exclusively among ILD patients. Data on other respiratory indications remain scarce, particularly for COPD, which nonetheless accounts for approximately 30-35% of lung transplantation procedures (264). This selection bias makes it difficult to generalize findings to other transplant indications, although two larger studies included some patients with COPD, cystic fibrosis, and pulmonary arterial hypertension (238, 239). Third, evidence outside the lung transplantation field is extremely limited. Only one moderate-sized cohort (248) and a few case series have reported on liver transplantation, while data on heart and kidney transplantation are virtually absent. Although TBDs are not typically associated with cardiac or renal disease, recipients of these organs may still harbor unrecognized TBDs and remain susceptible to complications from immunosuppressive drugs, particularly cytopenia. Still, the burden of TBD-related complications appears greatest in the lung transplantation setting, both because these recipients are enriched for TBDs and because post-lung transplant immunosuppression is significantly heavier.

Taken together, the current literature suggests that TBDs are associated with an increased risk of hematologic complications following lung transplantation, most notably thrombocytopenia, anemia, and neutropenia. Whether these translate into worse long-term outcomes, such as reduced survival or higher rates of CLAD, remains uncertain, as existing studies are likely underpowered to address such outcomes. By contrast, liver transplantation in TBD patients does not appear to confer the same hematologic burden. In fact, several reports describe improvements in anemia and thrombocytopenia post-transplant, possibly reflecting resolution of portal hypertension-related gastrointestinal bleeding and hypersplenism, although this explanation remains speculative.

Management of this at-risk group remains an area of uncertainty, both at the screening level and in the post-transplantation setting. In particular, the optimal timing for more invasive investigations of TBD-related conditions, such as bone marrow biopsy, is largely undefined. Furthermore, while immunosuppressive therapy is a recognized risk factor for hematological complications, it remains unclear whether proactive or reactive adjustments to the chronic immunosuppressive regimen should be applied, as they might participate in the increased rates of CLAD observed in some studies (238). The management proposals made by the authors are based on limited evidence and on their clinical experience and will likely evolve as knowledge advances regarding these disorders. Although awareness of TBD is increasing, particularly in the field of interstitial lung diseases, major knowledge gaps persist in the transplant context, underscoring the need for collaborative research to improve patient stratification and management.
